# Association of non-daily hookah tobacco smoking and cardiovascular disease-related exposure biomarkers among U.S. users: The Population Assessment of Tobacco and Health Study

**DOI:** 10.1016/j.pmedr.2023.102417

**Published:** 2023-09-13

**Authors:** Mary Rezk-Hanna, Amanda Adolfo, Umme Shefa Warda, Mary-Lynn Brecht, Neal L. Benowitz

**Affiliations:** a School of Nursing, University of California, Los Angeles, Los Angeles, CA, United States; b Divsion of Cardiology, Department of Medicine, University of California, San Francisco, San Francisco, CA, United States

**Keywords:** Hookah, Waterpipe, Cigarette, Tobacco smoking, Biomarkers, Cardiovascular disease risk, PATH study

## Abstract

Hookah smoking has grown to become a global tobacco epidemic. While cigarette smoking is a well-established cardiovascular disease (CVD) risk factor, the CVD risks of hookah smoking are unknown, particularly among regular U.S. adult hookah users who are predominantly non-daily users. Herein, we examined the association between hookah smoking and biomarkers of CVD risk among regular exclusive hookah smokers (*n* = 75), compared to regular exclusive cigarette smokers (*n* = 1773), dual hookah and cigarette smokers (*n* = 43) and never tobacco users (*n* = 757), using data from a nationally representative sample of adults from the Population Assessment of Tobacco and Health Study (2013–2014). Whereas 84% of cigarette smokers reported daily use, only 8% of hookah smokers reported daily use, with more than a third reporting monthly use. Adjusting for age and sex and as compared to exclusive cigarette smokers, exclusive hookah smokers had significantly lower geometric mean concentrations in serum sICAM-1 and urinary F2-isoprostane (p < 0.05). Although not statistically significant, a signal of increased oxidative stress was observed among hookah smokers as compared to never tobacco users (urinary F2-isoprostane). CVD-related harm biomarkers appear to be lower among hookah smokers than cigarette smokers. These findings represent patterns of hookah smoking predominantly shared among adult U.S. users who report non-daily occasional use and do not reflect solitary, daily use as is common in the Middle East. Future studies with longer exposure and longitudinal hookah use are warranted to explore the association between hookah smoking and CVD risk.

## 1 Introduction

Hookah (i.e., waterpipe) smoking is a centuries-old tobacco use method that has grown to become a global tobacco epidemic (Maziak et al., 2015). National representative data from Wave 1 (2013–2014) of the Population Assessment of Tobacco and Health (PATH) Study showed that 18.2% of adults 18–24 years of age reported current hookah use, comparable to 19.6% who reported cigarette use (Kasza et al., 2017). While cigarette smoking is a well-established cardiovascular disease (CVD) risk factor (Tsao et al., 2022), the CVD risks of hookah smoking are unknown, particularly among regular U.S. adult hookah tobacco users who are predominantly non-daily users. While studies have reported associations of regular hookah tobacco use with increased CVD risk, severity, and mortality (Ward et al., 2015, Sezavar and Bazargan, 2004, Sibai et al., 2014, Suwaidi et al., 2012), most studies are from the Middle East, where: (a) hookah is often a solitary activity smoked daily with the majority of users smoking several times/day; and/or (b) the characteristics of the smoked product (flavored tobacco, jurak, or tumbak) are not clearly identified (Fouad et al., 2016, Selim et al., 2013). These differences need to be considered carefully when extrapolating to CVD effects among U.S. users reporting occasional non-daily use.

To address these gaps, the present study investigates the association between hookah smoking and four serum biomarkers of inflammation, including high-sensitivity C-reactive protein (hs-CRP), interleukin-6 (IL-6), soluble intercellular adhesion molecule (sICAM-1), and fibrinogen, and one biomarker of oxidative stress (urinary levels of 8-isoprostane (F2-isoprostane)) as biomarkers of CVD risk in regular exclusive adult hookah smokers, compared to regular exclusive cigarette smokers, dual hookah and cigarette smokers and never-users of tobacco products, participating in the PATH Study (2013–2014). Nicotine intake was assessed using urinary total nicotine equivalents (TNE; sum of nicotine and six metabolites, including cotinine, 3-hydroxycotinine, nicotine *N*-oxide, cotinine *N*-oxide, nornicotine, and norcotinine) (Hukkanen et al., Mar 2005), and urinary cotinine.

## 2 Methods

Data were derived from the PATH study, an ongoing, nationally representative, longitudinal cohort study, conducted in 2013–2014 (Wave 1), to describe patterns of tobacco and alternative tobacco use and associated health outcomes among non-institutionalized residents aged ≥ 12 years in the U.S. (Hyland et al., 2017). The PATH data collection was conducted by Westat and approved by Westat’s institutional review board. Briefly, participants who completed Wave 1 interview questionnaire were asked to provide blood and urine specimens for analysis. For blood, phlebotomists visited participants at their home to administer a brief set of questions about use of tobacco products during the 3-day period prior to blood collection. The phlebotomist then collected the specimens, immediately placed in a Credo Cube shipper, to hold specimens between 2 °C and 8 °C and shipped overnight to the PATH Study biorepository for storage and processing. For urine, participants self-collected full-void urine specimens in 500 mL polypropylene containers, which were placed in a custom Credo Cube shipper, to hold specimens between 2 °C and 8 °C and shipped overnight to the PATH Study biorepository for storage and processing. Because serum biomarkers of inflammation, including hs-CRP, IL-6, sICAM-1, and fibrinogen, were only collected in the first wave of PATH, we used data from that wave (2013–2014) to examine the study aims. PATH study restricted-use data files are available at the National Addiction & HIV Data Archive Program (National Institute on Drug Abuse aUDoHaHS, Food and Drug Administration, Center for Tobacco Products, 2017).

Participants were divided into four groups: hookah smokers, cigarette smokers, dual hookah and cigarette smokers, and never-tobacco users. Hookah smokers were defined as those who reported being established users (ever smoked, smoked regularly, and smoke every day or some days), smoked at least once in the past 30 days, have not smoked or used any other tobacco product in the past 30 days and did not use nicotine replacement therapy (NRT) “today, yesterday, or the day before yesterday.” Cigarette smokers were defined as those who reported being established users (ever smoked, smoked > 100 cigarettes in lifetime, and smoke every day or some days), smoked at least once in the past 30 days, have not smoked or used any other tobacco product in the past 30 days and did not use NRT “today, yesterday, or the day before yesterday.” Dual hookah and cigarette users were defined as those who exclusively smoke hookah plus cigarettes, similar to the above criteria. Never tobacco users were defined as those who have never smoked or used any tobacco or nicotine product and had urinary cotinine < 10 ng/mL. All participants self-reported data on: age (18–24, 25–34, and >35 years old); sex (male, female); race/ethnicity (white non-Hispanic, other); marijuana use (current, never); education (some college, no college); annual income (<$25 000, 25 000– 49 999, >50 000); BMI (<18.5, 18.5–24.9, 25–29.9; ≥30); and CVD diagnoses (high blood pressure, high cholesterol, congestive heart failure, stroke, myocardial infarction or bypass surgery).

Analyses used survey weights with Fay’s variant to provide nationally representative statistics. Unadjusted geometric means with 95% confidence intervals were used to describe biomarkers of CVD risk. Weighted linear regression on log transformed biomarkers was used to compare groups adjusting for age and sex. As a sensitivity analysis, regressions were run adjusting for age, sex, race/ethnicity, marijuana use, education, income, BMI, and CVD diagnoses as well as for a subset of these covariates (age, sex, BMI, and CVD diagnosis). SAS Survey Procedures (V9.4) was used for all data analyses with significance level set at 0.05. Because analysis focused on deidentified data, it was exempted by the University of California, Los Angeles Institutional Review Board.

## 3 Results

Of the 2,648 participants, a total of 1773 were exclusive established cigarette smokers, 75 were exclusive established hookah smokers, 43 participants were dual hookah plus cigarettes smokers and 757 were identified as never tobacco users (Table 1). Both hookah smokers and dual hookah and cigarette smokers were predominantly young adults between 18 and 24 years of age (79% and 68%), as compared to 13% of participants in the cigarette smoker group (both p < 0.05). While BMI did not differ significantly between groups, 41% of exclusive cigarette smokers reported having CVD diagnoses, as compared to 11% of exclusive hookah smokers and 8% of dual hookah and cigarette smokers (both p < 0.05). Whereas 84% of cigarette smokers reported daily use, only 8% of hookah smokers reported daily use, with more than a third reporting monthly use. Nearly all (94%) hookah smokers reported sharing the same hookah pipe with others during smoking sessions. Among cigarette smokers, approximately a third of participants reported smoking 11–20 cigarettes per day (CPD). Hookah smokers reported first starting smoking hookah “fairly regularly” at 19.4 ± 0.5 years of age, while cigarette smokers reported first started smoking cigarettes “fairly regularly” at 17.8 ± 0.2 years of age. Cigarette smokers and dual users had substantially higher levels of nicotine exposure compared to exclusive hookah smokers (p < 0.05; Table 1). Adjusting for age and sex and as compared to exclusive cigarette smokers, exclusive hookah smokers had significantly lower geometric mean concentrations in serum sICAM-1 and urinary F2-isoprostane (p < 0.05). Although not statistically significant, a signal of increased oxidative stress was observed among exclusive hookah smokers as compared to never tobacco users (urinary F2-isoprostane). Exclusive cigarette smokers had consistently significantly higher levels of IL-6, sICAM-1, and cotinine, as compared to dual hookah and cigarette smokers, after adjusting for age and sex, BMI and CVD diagnoses (data not shown).

**Table 1:** Geometric mean estimates (95% confidence intervals) for smoking status associated with cardiovascular disease-related exposure biomarker concentrations among US non-institutionalized residents aged 12 years or older who participated in the Population Assessment of Tobacco and Health (PATH) Study: 2013–2014 .

***Biomarker***		***Hookah*** ***Smokers (Referent)*** ***N*** = ***75***	***Cigarette*** ***Smokers*** ***N*** = ***1773***	***Hookah*** + ***Cigarettes Smokers*** ***N*** = ***43***	***Never*** ***Tobacco Users*** ***N*** = ***757***
Inflammation biomarker	Serum hs-CRP, mg/L	1.24 (0.97–1.58)	1.79 (1.65–1.94)	0.83 (0.54–1.28)	1.45 (1.24–1.70)
	Adjusted for all covariates†	Ref.	−0.17 (0.13)	−0.42 (0.19)*	−0.39 (0.13)*
	Adjusted for age, sex, BMI,				
	CVD diagnoses	Ref.	−0.08 (0.13)	−0.40 (0.19)*	−0.33 (0.13)*
	Adjusted for age and sex	Ref.	−0.06 (0.15)	−0.45 (0.23)	−0.31 (0.15)*
Inflammation biomarker	Serum IL-6, pg/mL	1.14 (0.96–1.35)	1.70 (1.61–1.81)	0.94 (0.76–1.17)	1.35 (1.25–1.46)
	Adjusted for all covariates†	Ref.	0.03 (0.10)	−0.21 (0.13)	−0.16 (0.10)
	Adjusted for age, sex, BMI,				
	CVD diagnoses	Ref.	0.10 (0.09)	−0.18 (0.12)	−0.16 (0.09)
	Adjusted for age and sex	Ref.	0.10 (0.10)	−0.21 (0.14)	−0.16 (0.10)
Inflammation biomarker	Serum sICAM-1, ng/mL	206.08 (186.45– 227.77)	273.37 (262.79–284.37)	215.11 (191.61– 241.49)	212.64 (204.19–221.44)
	Adjusted for all covariates†	Ref.	0.15 (0.05)*	0.03 (0.08)	−0.10 (0.05)
	Adjusted for age, sex, BMI,				
	CVD diagnoses	Ref.	0.21 (0.06)*	0.04 (0.08)	−0.05 (0.05)
	Adjusted for age and sex	Ref.	0.21 (0.06)*	0.04 (0.08)	−0.05 (0.06)
Inflammation biomarker	Serum Fibrinogen, mg/dL	288.09 (265.82–312.23)	328.86 (319.90–338.07)	295.32 (265.06–329.03)	317.04 (310.28–323.95)
	Adjusted for all covariates†	Ref.	0.05 (0.04)	0.03 (0.05)	−0.002 (0.04)
	Adjusted for age, sex, BMI,				
	CVD diagnoses	Refs.	0.05 (0.04)	0.03 (0.06)	−0.001 (0.04)
	Adjusted for age and sex	Ref.	0.05 (0.04)	0.02 (0.06)	−0.0003 (0.04)
Oxidative stress biomarker	Urinary F2-isoprostane, pg/mg	469.18 (399.89–550.47)	572.23 (543.08– 602.95)	575.10 (446.40–740.90)	375.11 (345.62–407.12)
	Adjusted for all covariates†	Ref.	0.27 (0.09)*	0.15 (0.12)	−0.07 (0.09)
	Adjusted for age, sex, BMI,				
	CVD diagnoses	Ref.	0.33 (0.08)*	0.21 (0.13)	−0.07 (0.08)
	Adjusted for age and sex	Ref.	0.33 (0.09)*	0.20 (0.13)	−0.07 (0.09)
Biochemical measure of nicotine intake	Urinary Cotinine, ng/mL	10.49 (4.80–22.93)	2004.41 (1793.26–2240.41)	948.61 (418.74–2148.99)	0.29 (0.25–0.34)
	Adjusted for all covariates†	Ref.	5.12 (0.38)*	4.46 (0.52)*	−3.57 (0.39)*
	Adjusted for age, sex, BMI,				
	CVD diagnoses	Ref.	5.36 (0.39)*	4.54 (0.54)*	−3.43 (0.41)*
	Adjusted for age and sex	Ref.	5.36 (0.39)*	4.51 (0.54)*	−3.44 (0.41)*
Biochemical measure of nicotine intake	Urinary TNE, nmol/mL	6.84 (4.24–11.03)	50.90 (46.86–55.30)	29.68 (19.15– 46.00)	–
	Adjusted for all covariates†	Ref.	1.59 (0.22)*	1.46 (0.32)*	–
	Adjusted for age, sex, BMI,				
	CVD diagnoses	Ref.	1.64 (0.23)*	1.45 (0.34)*	–
	Adjusted for age and sex	Ref.	1.62 (0.22)*	1.41 (0.33)*	–

*Indicates significant difference from referent. Hs-CRP indicates high sensitivity C-reactive protein; IL-6, interleukin-6; sICAM, soluble intercellular adhesion molecule; and TNE, total nicotine equivalents. Data are shown as unweighted Ns and weighted geometric mean (95% CI); and regression coefficient (standard error), comparing hookah to other tobacco and never tobacco users. Note that the first row for biomarkers represents the weighted geometric means (95% CI), whereas the remaining rows include comparisons of exclusive hookah smokers (the reference group) to each other smoker group with coefficients (standard errors) from linear regression of log transformed biomarker and that each of these rows includes different sets of covariates in the models. †Adjusting for age, sex, race/ethnicity, marijuana use, education, income, BMI, and CVD diagnosis. (-) indicates no data available.

## 4 Discussion

Utilizing nationally representative data, the present study extends findings from acute exposure studies, (Bhatnagar et al., 2019) to document lower CVD-related harm biomarkers resulting from regular hookah smoking, as compared to cigarette smoking. Our findings, however, should be interpreted with caution, primarily due to the total exposure duration and intermittent use patterns reported by study participants of varying ages. In our study, as compared to 84% of cigarette smokers who reported daily use, where a third reported smoking 11–20 CPD, only 8% of hookah smokers reported daily use and nearly all (94%) reported sharing the same waterpipe with others. The extent of tobacco smoke exposure as indicated by urinary nicotine equivalents and cotinine was much lower with hookah use compared to cigarette smoking. Notably, the age of exclusive hookah smokers was much lower compared to exclusive cigarette smokers. Since CVD-related harms of cigarette as well as hookah smoking are likely to be cumulative, it is possible that the exposure among the younger hookah smoking group has not been long, as well as frequent enough to affect CVD risk. Moreover, the complexity of cardiovascular-related responses to hookah smoking should be considered, because of the wide mixture of constituents resulting not only from tobacco combustion but also from fruit and charcoal combustion, with some constituents inducing different and potentially opposing effects (Rezk-Hanna et al., 2020). Of particular interest is carbon monoxide—a key charcoal combustion product that has been shown to increase to a nearly 8-fold greater extent after a single hookah smoking session, as compared to after smoking a cigarette— (Bhatnagar et al., 2019). Carbon monoxide has been shown to exert potent anti-inflammatory effects (Otterbein, 2002). In-vitro studies demonstrate a protective role of carbon monoxide by inhibiting C-reactive protein expression (Chung et al., 2011), and pharmacological application of carbon monoxide releasing molecules provide anti-inflammatory effects by interfering with nuclear factor-κB activation (Cepinskas et al., 2008). Our findings of lower levels of some inflammatory biomarkers among exclusive hookah and dual hookah plus cigarette smokers compared to exclusive cigarette smokers might be explained by high exposure to carbon monoxide in young adult hookah smokers, which could counter the pro-inflammatory effects of smoke inhalation. Putatively via its anti-inflammatory actions, carbon monoxide appears to reduce oxidative stress at lower levels, but at higher levels, when associated with tissue ischemia, carbon monoxide increases oxidative stress (Ryter et al., 2007, Korbut et al., 2020). Unfortunately, no PATH data on carbon monoxide levels were available. It is plausible that carbon monoxide levels could dampen oxidative stress and therefore reduce urinary F2-isoprostane. Because exposure to carbon monoxide reduces hemoglobin oxygen-carrying capacity, future studies investigating the potential health implications of chronic carbon monoxide exposure among exclusive hookah smokers who show less inflammation and oxidative stress than cigarette smokers, are warranted. It is noteworthy to mention that no PATH data were available about study participants’ diet or physical activity, both confounding factors that may have influenced the magnitude of the association between tobacco use and biomarkers of CVD risk.

To our knowledge, our study is among the first to explore the association between hookah smokers and biomarkers of inflammation and oxidative stress among nationally represented established adult users. Our findings suggest that CVD-related harm biomarkers are lower among exclusive hookah smokers than cigarette smokers. These findings represent patterns of hookah tobacco smoking predominantly shared among young adult U.S. users who report non-daily occasional use and do not reflect solitary, daily use as is common in the Middle East. Future extensive studies with longer exposure and longitudinal hookah use are warranted to effectively explore the association between hookah smoking and CVD risk.

## Funding

This study was supported, in part, by the National Heart, Lung, and Blood Institute of the National Institutes of Health (grant U54 HL 147127) to Dr. Benowitz and (grant 1R01HL152435-01A1) to Dr. Rezk-Hanna.

## CRediT authorship contribution statement

**Mary Rezk-Hanna:** Conceptualization, Methodology, Formal analysis, Writing – original draft, Supervision, Funding acquisition. **Amanda Adolfo:** Data curation, Writing – original draft, Writing – review & editing, Visualization. **Umme Shefa Warda:** Visualization, Investigation, Software, Formal analysis, Writing – review & editing. **Mary-Lynn Brecht:** Methodology, Software, Formal analysis, Writing – review & editing. **Neal L. Benowitz:** Conceptualization, Methodology, Writing – review & editing, Funding acquisition.

## Declaration of Competing Interest

The authors declare the following financial interests/personal relationships which may be considered as potential competing interests: Dr. Benowitz consults with pharmaceutical companies that market or are developing smoking cessation medications and has been a paid expert witness in litigation against tobacco companies. All other authors disclose no conflicts of interest.

## Data Availability

Data will be made available on request.

## References

[b0005] Bhatnagar A., Maziak W., Eissenberg T., Ward K.D., Thurston G., King B.A., Sutfin E.L., Cobb C.O., Griffiths M., Goldstein L.B., Rezk-Hanna M. (2019). Water pipe (hookah) smoking and cardiovascular disease risk: A scientific statement from the American Heart Association. Circulation.

[b0010] Cepinskas G., Katada K., Bihari A., Potter R.F. (2008). Carbon monoxide liberated from carbon monoxide-releasing molecule CORM-2 attenuates inflammation in the liver of septic mice. Am. J. Physiol. Gastrointest. Liver Physiol..

[b0015] Chung J., Shin D.-Y., Zheng M., Joe Y., Pae H.-O., Ryter S.W., Chung H.-T. (2011). Carbon monoxide, a reaction product of heme oxygenase-1, suppresses the expression of C-reactive protein by endoplasmic reticulum stress through modulation of the unfolded protein response. Mol. Immunol..

[b0020] Fouad H., Awa F.E., Naga R.A.E., Emam A.H., Labib S., Palipudi K.M., Andes L.J., Asma S., Talley B. (2016). Prevalence of tobacco use among adults in Egypt, 2009. Glob. Health Promot..

[b0025] Hukkanen J., Jacob P., Benowitz N.L. (2005). Metabolism and disposition kinetics of nicotine. Pharmacol. Rev..

[b0030] Hyland A., Ambrose B.K., Conway K.P., Borek N., Lambert E., Carusi C., Taylor K., Crosse S., Fong G.T., Cummings K.M., Abrams D., Pierce J.P., Sargent J., Messer K., Bansal-Travers M., Niaura R., Vallone D., Hammond D., Hilmi N., Kwan J., Piesse A., Kalton G., Lohr S., Pharris-Ciurej N., Castleman V., Green V.R., Tessman G., Kaufman A., Lawrence C., van Bemmel D.M., Kimmel H.L., Blount B., Yang L., O'Brien B., Tworek C., Alberding D., Hull L.C., Cheng Y.-C., Maklan D., Backinger C.L., Compton W.M. (2017). Design and methods of the Population Assessment of Tobacco and Health (PATH) Study. Tob. Control.

[b0035] Kasza K.A., Ambrose B.K., Conway K.P., Borek N., Taylor K., Goniewicz M.L., Cummings K.M., Sharma E., Pearson J.L., Green V.R., Kaufman A.R., Bansal-Travers M., Travers M.J., Kwan J., Tworek C., Cheng Y.-C., Yang L., Pharris-Ciurej N., van Bemmel D.M., Backinger C.L., Compton W.M., Hyland A.J. (2017). Tobacco-product use by adults and youths in the United States in 2013 and 2014. N. Engl. J. Med..

[b0040] Korbut E., Brzozowski T., Magierowski M. (2020). Carbon monoxide being hydrogen sulfide and nitric oxide molecular sibling, as endogenous and exogenous modulator of oxidative stress and antioxidative mechanisms in the digestive system. Oxid. Med. Cell. Longev..

[b0045] Maziak W., Taleb Z.B., Bahelah R., Islam F., Jaber R., Auf R., Salloum R.G. (2015). The global epidemiology of waterpipe smoking. Tob. Control.

[b0050] National Institute on Drug Abuse aUDoHaHS, Food and Drug Administration, Center for Tobacco Products. 2017. Population Assessment of Tobacco and Health (PATH) Study [United States] Biomarker Restricted-Use Files (ICPSR36840). Ann Arbor, MI: Inter-university Consortium for Political and Social Research.

[b0055] Otterbein L.E. (2002). Carbon monoxide: innovative anti-inflammatory properties of an age-old gas molecule. Antioxid. Redox Signal..

[b0060] Rezk-Hanna M., Nelson M.D., Rader F., Benowitz N.L., Rosenberry R., Chang L.C., Li N., Tashkin D.P., Elashoff R.M., Victor R.G. (2020). Peripheral blood flow changes to cutaneous and muscular beds in response to acute hookah smoking. Am. J. Cardiol..

[b0065] Ryter S.W., Kim H.P., Hoetzel A., Park J.W., Nakahira K., Wang X., Choi A.M.K. (2007). Mechanisms of cell death in oxidative stress. Antioxid. Redox Signal..

[b0070] Selim G.M., Fouad H., Ezzat S. (2013). Impact of shisha smoking on the extent of coronary artery disease in patients referred for coronary angiography. Anadolu Kardiyol. Derg..

[b0075] Sezavar S.A., Bazargan H. (2004). A comparative study of plasma fibrinogen among hookah smokers, cigarette smokers and non-smokers. Iran. Heart J..

[b0080] Sibai A.M., Tohme R.A., Almedawar M.M., Itani T., Yassine S.I., Nohra E.A., Isma'eel H.A. (2014). Lifetime cumulative exposure to waterpipe smoking is associated with coronary artery disease. Atherosclerosis.

[b0085] Suwaidi J.A., Zubaid M., El-Menyar A.A., Singh R., Asaad N., Sulaiman K., Mahmeed W.A., Al-Shereiqi S., Akbar M., Binali H.A.A. (2012). Prevalence and outcome of cigarette and waterpipe smoking among patients with acute coronary syndrome in six Middle-Eastern countries. Eur. J. Prev. Cardiol..

[b0090] Tsao C.W., Aday A.W., Almarzooq Z.I., Alonso A., Beaton A.Z., Bittencourt M.S., Boehme A.K., Buxton A.E., Carson A.P., Commodore-Mensah Y., Elkind M.S.V., Evenson K.R., Eze-Nliam C., Ferguson J.F., Generoso G., Ho J.E., Kalani R., Khan S.S., Kissela B.M., Knutson K.L., Levine D.A., Lewis T.T., Liu J., Loop M.S., Ma J., Mussolino M.E., Navaneethan S.D., Perak A.M., Poudel R., Rezk-Hanna M., Roth G.A., Schroeder E.B., Shah S.H., Thacker E.L., VanWagner L.B., Virani S.S., Voecks J.H., Wang N.-Y., Yaffe K., Martin S.S. (2022). Heart disease and stroke statistics-2022 update: A report from the American Heart Association. Circulation.

[b0095] Ward K.D., Ahn S., Mzayek F., Al Ali R., Rastam S., Asfar T., Fouad F., Maziak W. (2015). The relationship between waterpipe smoking and body weight: population-based findings from Syria. Nicotine Tob. Res..

